# A Comparison of Centroid Tracking and Image Phase for Improved Optokinetic Nystagmus Detection

**DOI:** 10.3390/jemr19010012

**Published:** 2026-01-26

**Authors:** Jason Turuwhenua, Mohammad Norouzifard, Zaw LinTun, Misty Edmonds, Rebecca Findlay, Joanna Black, Benjamin Thompson

**Affiliations:** 1 Auckland Bioengineering Institute, The University of Auckland, Auckland 1142, New Zealand; m.norouzifard@auckland.ac.nz (M.N.); zaw.lin.tun@auckland.ac.nz (Z.L.); 2 School of Optometry and Vision Science, The University of Auckland, Auckland 1142, New Zealand; r.findlay@auckland.ac.nz (R.F.); j.black@auckland.ac.nz (J.B.); 3 Iwi United Engaged Ltd., Drury 2579, New Zealand; misty@iue.net.nz; 4 School of Optometry and Vision Science, The University of Waterloo, Waterloo, ON N2L 3G1, Canada; ben.thompson@uwaterloo.ca; 5 Centre for Eye and Vision Research, 17W Hong Kong Science Park, Hong Kong; 6 Liggins Institute, The University of Auckland, Auckland 1142, New Zealand

**Keywords:** image phase, centroid tracking, optokinetic nystagmus, motion microscopy

## Abstract

Optokinetic nystagmus (OKN) is an involuntary sawtooth eye movement that occurs in the presence of a drifting stimulus. Our experience is that low-amplitude/short-duration OKN can challenge the limits of our commercially available Pupil Neon eye-tracker, leading to false negative OKN detection results. We sought to investigate whether such instances could be remediated. We compared automated OKN detection using: (1) the gaze signal from the Pupil Neon (OKN-G), (2) centroid tracking (OKN-C), and (3) an image-phase-based “motion microscopy” technique (OKN-MMIC). The OKN-C and OKN-MMIC methods were also tested as a remediated step after a negative OKN-G result (OKN-C-STEP, OKN-MMIC-STEP). To validate the approaches adults (*n* = 22) with normal visual acuity was measured whilst viewing trials of an OKN induction stimulus shown at four levels of visibility. Confusion matrices and performance measures were determined for a “main” dataset that included all methods, and a “retest” set, which contained instances where centroid tracking failed. For the main set, all tested methods improved upon OKN-G by Matthew’s correlation coefficient (0.80–0.85 vs. 0.76), sensitivity (0.89–0.95 vs. 0.85), and accuracy (0.91–0.93 vs. 0.88); but only OKN-C yielded better specificity (0.90–0.96 vs. 0.95). For the retest set, MMIC and MMIC-STEP methods consistently improved upon the performance of OKN-G across all measures.

## 1. Introduction

Optokinetic nystagmus (OKN) is a reflexive, involuntary eye movement that occurs in response to a drifting stimulus. It appears as a distinctive eye beating, or equivalently, as sawtooth displacements in eye tracking displacement data. These eye-motions occur as the eye repeatedly tracks the stimulus (which is the slow phase, SP, of OKN) and then resets back (the quick phase, QP, of OKN) to maintain fixation on the moving pattern [1].

Our interest in OKN has been its potential to objectively measure visual acuity (VA), the quantitative measure of the ability to see fine details, typically measured by reading aloud the diminishing letters on an eye chart. Estimation of VA can be challenging in patients such as pre-verbal children [2] or adults who may struggle to reliably complete standard visual acuity tests [3]. Currently, OKN is utilized for subjective VA assessment in these individuals by rotating a striped hand-held drum in front of the eye, and the visibility of the stimulus is inferred from the presence/absence of the OKN response as judged by the clinician. The angular size of the stripe that induced OKN is an estimate of VA. Crucially, the optokinetic drum does not require an explicit response from the patient because OKN is reflexive [4]. On the other hand, this approach yields a gross and highly subjective measure of visual performance. Objective/automated methods of utilizing OKN for VA assessment could significantly improve vision assessment in patients who cannot complete standard VA testing protocols [2,5].

Methods for the automated and objective analysis of nystagmus-like signals have been investigated [6,7,8,9,10] and correlations between VA found by OKN and standard VA have been reported previously, based on subjective observation of the eye or eye tracking data [11,12,13,14,15,16]. Recent work combined automated analysis of the eye-tracker signal for objective estimation of VA and contrast sensitivity using research-grade eye tracking [17,18,19,20]. Methods for accessible mobile-based measurement of OKN have been recently reported using machine learning (ML) [21,22] as well as cheaper hardware solutions [11]. Machine learning methods have also been proposed for the estimation of eye signals measured using mobile phones, as well as the classification of OKN-like signals, [23,24,25] enabling the potential for lower cost and also ubiquitous measurement.

In moving towards accessible measurement, such as in community and clinical settings, accurate measurement of OKN is paramount [20]. This requires a device that can deal with the natural variations between participants (e.g., eye positioning, eyelashes), the environmental conditions (e.g., room lighting), and can be deployed readily when time is pressured. For this reason, we employed the Pupil Neon (Pupil Labs, https://pupil-labs.com/ (accessed on 1 December 2025)) eye-tracker in our recent OKN research. This system is ML-based, trained on eye tracking data, and can estimate eye gaze from eye camera images without specific calibration procedures. Importantly, this system avoids session-specific calibration and can be deployed “out-of-the-box” across a range of environmental conditions [26].

**Figure 1:**
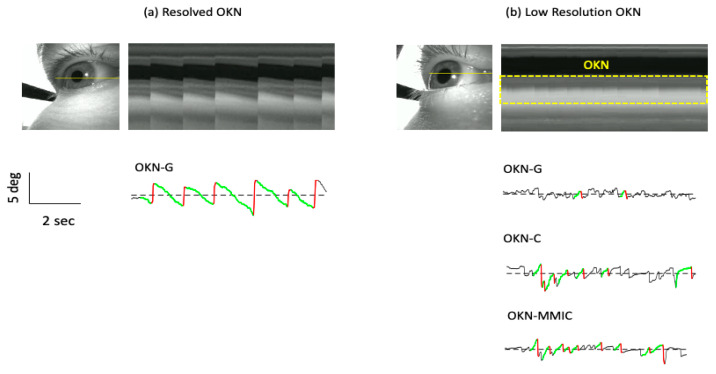
Results of automated OKN detection. (**a**) An example of a stereotypical well-resolved OKN found from a single eye. The figure shows an image from the eye camera with a horizontal line indicating the source of pixels used to form the cross-sectional view. Below the cross-section is the gaze signal obtained from the Pupil Neon eye-tracker, along with automated OKN detection results. The 1D signals are shown with detected slow phases (green) amd quick phases (red) superimposed. (**b**) Low resolution OKN. In this case, small amplitude/high frequency oscillations were seen in the eye camera video (see cross-sectional view and area within the dashed line). However, these oscillations are not readily apparent using OKN-G. Output from the OKN-C and OKN-MMIC methods reveals additional instances of OKN.

This work was motivated by our recent experience with the Pupil Neon eye-tracker and its application to the measurement of OKN. Figure 1a shows an example of a stereotypical “stare” type OKN [27,28] which refers to the condition in which the observer maintained gaze on the center of the OKN inducing stimulus. This example presents results found by our own OKN detection methods applied to the eye gaze signal [29] referred to hereinafter as OKN by gaze or OKN-G. The result seen here is highly regular in nature (~0.8 Hz) and readily visible (~4 deg). The regularity is confirmed by the cross-section of eye image profiles corresponding to the horizontal yellow line on the image of the eye in Figure 1a. The repeated clear horizontal sliding motion of the eye (SP), followed by the reset (QP) that defines OKN was observed in the eye camera video.

However, we also found the case illustrated by Figure 1b in which the eye’s oscillations occur on a smaller scale (lower amplitude/shorter duration), resulting in false-negative (FN) detections. It is seen in this instance that OKN was poorly resolved in the output signal, leading to sporadic OKN detection as confirmed by the relative absence of OKN-G detection results. Visual inspection of the cross-section of eye camera video confirms an underlying lower amplitude (~1 deg) and more rapid beating (~1.8 Hz) of the eye.

In this work, we tested the idea that an improved eye signal could be extracted directly from the eye camera videos. For this purpose, we used two alternate methods of signal estimation (followed by automated OKN detection) that were compared against OKN-G. Firstly, we implemented automated OKN detection based on a simple pupil tracking algorithm applied to the eye camera videos (OKN-C). The pupil tracking algorithm thresholded the image to produce a pupil estimation, and the displacement was the centroid estimate. Centroid tracking is fundamental to the majority of published methods for the estimation of eye motion using infrared illumination of the eye [30,31]. An example result obtained for OKN-C is shown in Figure 1b.

Secondly, we tested automated OKN detection based on the *motion microscope* technique (OKN-MMIC) for the recovery of the signal from video [32]. That method uses local image phase to estimate regional motion displacement, and it has found application mainly in the estimation of small periodic oscillations in 2D vibrational analysis applications. Indeed, several vibrational studies [32,33,34] established excellent tracking performance across amplitudes ranging from several pixels (2–3) to a few thousandths of a pixel. More recently, the approach was extended for 3D use for the quantitative analysis of small pulsatile motions in MRI images [35] where it quantified motions on the order of 0.01 voxels. However, it has not been applied for quantitative eye tracking to date, as far as we are aware, although the potential of phase for amplifying and enhancing the presence of saccadic eye movements was demonstrated in related work, albeit qualitatively, using high-speed RGB camera video [36,37,38]. The details of the method we employed will be explained in the Section 2 to follow, and an example result obtained for OKN-MMIC is shown in Figure 1b.

The general concept of optical flow as a method for eye tracking has been investigated by several authors [39,40,41,42] but that work focused on gross rather than subtle eye displacements, and used intensity-based optical flow rather than phase [43,44]. The standard intensity optical flow equation estimates displacement by integration and up to a constant, and in this work, it will be seen that the proposed approach estimates displacement directly from a decomposition of each video frame, thereby avoiding this additional step. On the other hand, optical flow methods are inherently Eulerian, so that data is read from a desired location in the image rather than the location of a moving feature [45]. This potentially avoids some of the pitfalls of feature-based/Lagrangian methods, which require sufficiently clean images and/or robust algorithms to track a feature, such as the pupil, for example. Such methods require explicit mitigations to deal with confounding issues, such as poor lighting, and intrusions, such as eyelashes and shadows, whereas optical flow methods are less sensitive to these confounders [42].

The paper is organized as follows: we start by introducing the motion microscope method. We then describe two experiments. In the first experiment, we compare OKN-G to (1) centroid tracking-based OKN estimation (OKN-C), (2) motion microscope method-based OKN estimation (OKN-MMIC), and the same methods applied only to datasets in which OKN was present and the OKN-G method produced a false negative (OKN-C-STEP/OKN-MMIC-STEP). This latter strategy aimed to enhance the instances of poorly resolved OKN, where OKN was not detected, rather than serve as a replacement method. In a second experiment, we tested the motion microscope methods (OKN-MMIC/OKN-MMIC-STEP) on a smaller set of eye camera videos that were removed from the main dataset because the centroid tracking method failed to yield quality data in these instances. These experiments demonstrate that motion microscopy can be effective in situations where a standard eye tracking algorithm fails. The methods just introduced will be described again in more detail in the following sections, and for convenience, the methods and a short summary description have been added to the abbreviations list.

## 2. Background

### 2.1. The Motion Microscope (MMIC)

**Figure 2:**
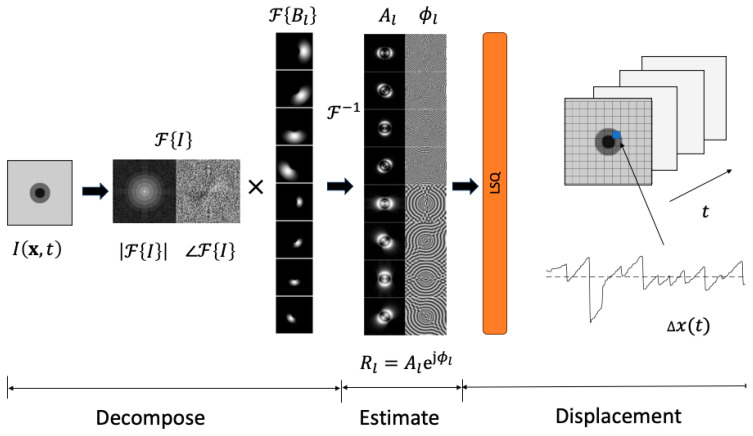
The method takes the discrete Fourier transform of the original image $I\left(x,t\right)$ and multiplies it by a bank of filters $F\left\{B_{l}\right\}$ that each selects for a particular frequency and orientation. The local phase $\phi_{l}(x,t)$ and amplitude $A_{l}(x,t)$ is estimated by taking the inverse Fourier Transform of the result. Displacement is determined by a weighted least squares solution of a phase-based optical flow equation. The blue square indicates the pixel from which the displacement signal is taken.

The “motion microscope” estimates displacement from local image amplitude and phase [32]. The method proceeds in three steps summarized by Figure 2: (1) *image decomposition*: a given image frame is used to calculate a set of frequency-domain images that each represent a specific frequency band and orientation combination, or *level*, (2) *amplitude and phase estimation*: the resulting frequency-domain images found for each level, are found by the inverse discrete Fourier transform to yield complex spatial-domain images from which amplitude and phase are extracted, (3) *displacement estimation*: displacement is determined by a weighted least squares over phase and amplitude found for each level. More detail for each process is provided in the following sections.

### 2.2. Image Decomposition

The image decomposition in the frequency domain is performed by masks $F\left\{B_{l}\right\}$ that each represents polar regions (in frequency and orientation) of frequency space. Each mask is multiplied by the discrete Fourier transform of the image, denoted here by $F\{I\left(x,t\right)\}$.

$$F\{R_{l}(x,t)\}=F\{I\left(x,t\right)\}F\{B_{l}\},$$

Wadhwa et al. used a steerable pyramid (SP) decomposition [46] to achieve the partitioning by $F\{B_{l}\}$. That approach first divides frequency space into a high-pass and low-pass sub-band. The low-pass sub-band is divided into oriented band-pass sub-bands, and a lower-pass sub-band (found by down-sampling by a factor of two if the decomposition is “full octave”). The procedure is repeated recursively until the desired number of levels is reached. Examples of $F\left\{B_{l}\right\}$ are shown in Figure 2 for two scales and four orientations (a total of eight levels).

### 2.3. Amplitude and Phase Estimation

Crucially, the form of $F\left\{B_{l}\right\}$ and specifically, the absence of conjugate symmetry results in a set of complex images $R_{l}(x,t)$, found by the inverse discrete Fourier transform, given by

$$R_{l}(x,t)=A_{l}(x,t)e^{j\phi_{l}(x,t)}$$

This image in the complex spatial domain contains local estimates of amplitude $A_{l}(x,t)$ and phase $\phi_{l}(x,t)$ for the image, $I_{l}\left(x,t\right)$. Collier and Dare [33] motivated this process by noting the close similarity of this method to the 1D Hilbert transform, in which the negative frequencies are suppressed to yield the 1D analytic signal, from which the instantaneous amplitude and phase are then determined.

### 2.4. Least Squares Estimation

The motion microscope assumes that motion is encoded by the iso-contours of the *unwrapped* phase $\phi_{l}\left(x,t\right)$ [47],

$$\phi_{l}\left(x,t\right)=c$$

where the unwrapping refers to the removal of jump artifacts that occur because of the phase estimation process. If we define the phase relative to a start time *t* = 0,

$$\Delta \phi_{l}\left(x,t\right):=\phi_{l}\left(x,t\right)-\phi_{l}\left(x,0\right)$$

Applying the small displacement assumption to the above gives the following, after a first-order Taylor expansion,

$$\Delta \phi_{l}\left(x,t\right)\approx \left(\frac{\partial \phi_{l}\left(x,t\right)}{\partial x},\frac{\partial \phi_{l}\left(x,t\right)}{\partial y}\right)\cdot (\Delta x,\Delta y)$$

We then find the displacement d=(Δx,Δy) relative to the starting time, as the solution to the weighted least squares problem,

$$e^{2}\left(x,t\right)=\underset{\Delta x,\Delta y}{\arg min}\sum_{i}\;\rho_{l}^{2}(x,t){\left[\left(\frac{\partial \phi_{l}\left(x,t\right)}{\partial x},\frac{\partial \phi_{l}\left(x,t\right)}{\partial y}\right)\cdot (\Delta x,\Delta y)-\Delta \phi_{r_{i},\theta_{i}}\right]}^{2}$$

which gives the (weighted) normal equations in matrix form

$$A^{T}W^{2}Ad=A^{T}W^{2}b$$

where

$$\begin{array}{c} \begin{array}{cc} A=\left[\begin{array}{cc} \frac{\partial \phi_{l}\left(x,t\right)}{\partial x} & \frac{\partial \phi_{l}\left(x,t\right)}{\partial y}\\ \vdots & \vdots \\ \frac{\partial \phi_{L}\left(x,t\right)}{\partial x} & \frac{\partial \phi_{L}\left(x,t\right)}{\partial y} \end{array}\right], & b=\left[\begin{array}{cc} \Delta \phi_{l}\left(x,t\right)\frac{\partial \phi_{l}\left(x,t\right)}{\partial x} & \Delta \phi_{l}\left(x,t\right)\frac{\partial \phi_{l}\left(x,t\right)}{\partial y}\\ \vdots & \vdots \\ \Delta \phi_{L}\left(x,t\right)\frac{\partial \phi_{L}\left(x,t\right)}{\partial x} & \Delta \phi_{L}\left(x,t\right)\frac{\partial \phi_{L}\left(x,t\right)}{\partial y} \end{array}\right] \end{array}\\ W^{2}=\left[\begin{array}{ccc} r_{1}^{2}(x_{1},t) & & \\ & \ddots & \\ & & r_{L}^{2}(x_{N},t) \end{array}\right] \end{array}$$

The solution is

$$d={\left(A^{T}W^{2}A\right)}^{-1}A^{T}W^{2}b$$

In order that the square matrix $A^{T}W^{2}A$ be invertible, its rank needs to be equal to the number of rows (or columns). This requires that the number of levels L≥2 whereas in this work, the number of levels used was 20. Finally, it is noted that the phase-based optical flow method described here does not involve estimation of a time derivative, and in this respect, it differs markedly from the standard optical flow equation [44].

## 3. Materials and Methods

### 3.1. Participants

The study was conducted in accordance with the Declaration of Helsinki and approved by the University of Auckland Human Ethics Committee (UAHPEC20318). Informed consent was obtained from all subjects involved in the study. Participants were adults (*n* = 22, mean age 24 years) who had normal vision (20/20 or better) as determined by the Electronic Visual Acuity (EVA) Tester (Emmes, USA) and no evidence of ocular motility problems. Participants were assessed monocularly with the non-viewing eye occluded. All measurements were conducted in a laboratory at the University of Auckland, Auckland, or in a community setting in Ngāruawahia, New Zealand.

### 3.2. Stimulus

The stimulus was presented on a Samsung Galaxy S8 Ultra (https://www.samsung.com/) (accessed on 1 December 2025), which subtended 11.4 × 6.4 degrees horizontally at a 70 cm test distance. The OKN induction stimulus consisted of a field of horizontally drifting disks that moved consistently over 8 s trials at a speed of 5 deg/s. The field size and velocity used were consistent with previous studies of small-field OKN [48,49]. The stimulus was presented with a leftward or rightward motion that was randomized across trials at decreasing sizes of 0.5, 0.3, 0.1 logMAR, and no stimulus (with 3 repeats per logMAR for a total of 12 trials). The variation in stimulus size and presence altered the visibility of the stimulus and, therefore, induced full, partial, or no OKN responses. The number of trials within an experiment was designed to be limited to account for the fact that the protocol was designed for community deployment.

### 3.3. Participant Instructions

Participants were instructed to look at the display and maintain their gaze on the center of the screen during an experiment, whilst wearing eye-tracking glasses. The instruction was designed to induce “stare” OKN, which presents as a rapid and regular involuntary movement of the eye, as opposed to “look” OKN, which involves a slower, voluntary following of the stimulus [27,28]. Participants were asked to keep both eyes open, even though only one eye was tested at a time. Although the fellow eye was completely occluded, OKN is a yoked eye movement, and, therefore, the occluded eye moved in concert with the tested eye.

### 3.4. Eye Tracking

The Pupil Neon eye-tracker comprises wearable glasses with eye cameras mounted in the nose bridge. Gaze displacement signals and eye camera videos were streamed by the proprietary Neon Companion app software (Version 2.9.0-prod, Pupil Labs) to a PC for offline analysis. Gaze data was reported in degrees, and the sampling rate was 200 Hz. Eye camera videos were split into both left- and right-eye videos offline (192 × 192 pixels per frame). These videos were then processed individually to extract signals directly for each eye, using methods to be described below.

### 3.5. Ground Truth Assessment

**Figure 3:**
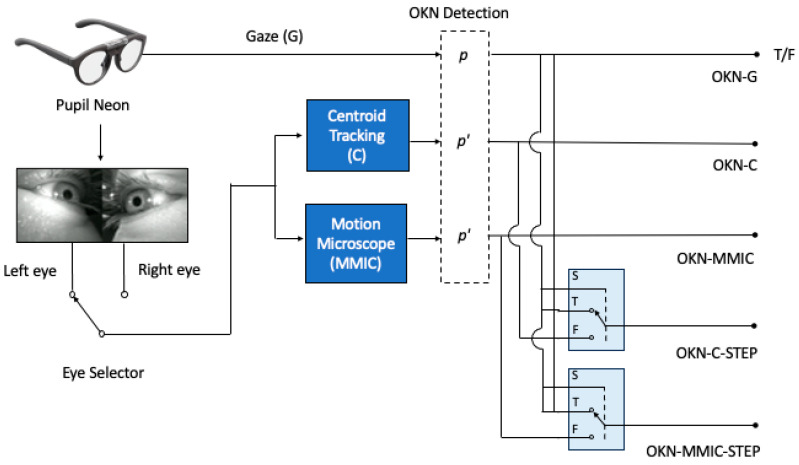
A description of the methods compared. The gaze (G) obtained from the Pupil Neon eye tracking glasses was passed through the OKN detector (with parameters *p*) to yield a per-trial OKN present (T)/absent (F) result (OKN-G). Four additional methods were applied to individual left- and right-eye camera videos. The OKN-C and OKN-MMIC methods were found by applying OKN detection (with parameters *p′*) to the C and MMIC-derived signals, respectively. The OKN-C-STEP and OKN-MMIC-STEP methods used the OKN-C/OKN-MMIC results *only* if OKN-G returned a not present result. The selection is represented by the two light blue multiplexing units, each driven by the input selector S, connected to the OKN-G line.

A manual ground truth assessment of OKN presence/absence was performed by three reviewers experienced in assessing OKN. The review used eye camera videos for each trial (which are obtained as side-by-side images of the two eyes, as shown in Figure 3) along with gaze displacement signals in angular units from the Pupil Neon, with the results of baseline OKN detection superimposed on that signal. Trials were labeled as containing OKN or not by the review process.

### 3.6. Eye Camera-Based Video Tracking

Eye camera displacement signals were determined using centroid tracking and the motion microscope methods applied directly to all available left/right-eye camera videos.

#### 3.6.1. Centroid Tracking (C)

Our implementation started by applying thresholding of the grayscale intensity to determine a binary image within a manually set region of interest. This step provided binary blobs/candidate pupils, which were closed by finding the convex hull and standard image-filling methods. Candidates were then thresholded by their equivalent diameter and circularity. The largest consistent pupil candidate was chosen as the pupil estimate, and the center of mass was calculated to give a positional estimate of the pupil center for a given frame.

The parameters of the centroid tracker (region of interest, intensity threshold, circularity, and equivalent diameter) were tuned for each participant’s eyes to ensure the pupil was optimally detected. This manual tuning mitigated the effect of distractors in the background of the image, which could reduce the quality of pupil segmentation. If no pupil estimate was found for a given frame, it was labeled as missing. Pupil estimation was run for all video frames of all the tested videos. Any videos with more than 30% data loss were categorized as “low data”.

#### 3.6.2. Motion Microscope Method (MMIC)

For this work, we used an implementation of the motion microscope method [32] provided by the authors of that work at: https://people.csail.mit.edu/nwadhwa/motion-microscope/ (accessed on 1 December 2025). The method was applied to left and right eye camera videos using a 20-level CSP (5 spatial scales × 4 orientations) with median filtering applied to the phase signal. Displacement in pixels was extracted from a point located nasal to the pupil and in the region of the limbal edge. This was estimated by using the mean pupil location determined by the centroid tracker, and a horizontal offset found for each participant to estimate the limbal position. The motion signal at this measurement point was averaged from the motion signal at neighboring positions (on a 5 × 5 pixel grid centered on this point).

### 3.7. Automated OKN

As noted already, automated OKN detection was applied to the gaze displacement signal extracted from the eye-tracker (the baseline assessment) and to signals extracted from the individual eye camera videos. A previously published method was used to determine the presence/absence of OKN in both cases [29]. In brief, this method determines OKN as triangular “sawteeth” consisting of onset, peak, and end points. The concept of the approach is to look for ramping in the *known* direction of the OKN induction stimulus, followed by a quick reset in the opposite direction. Any candidate OKN is tested for consistency and then thresholded on amplitude, duration, and velocity to determine its validity. Single sets of best OKN parameters were found empirically for the gaze method and the C and MMIC methods. It is noted that displacements for the latter two methods were in pixel units (not angular) and were therefore susceptible to camera distance and positioning, as well as the dimensions of the eyes encountered in the images.

OKN was signaled in a trial/video if repeating sawtooth detections (2 or more consecutive) were found, or if three or more isolated sawtooth features were present in a trial. This first criterion has been applied previously [19,42], and we refer to it here as the repeated OKN criterion. The second was recently incorporated to mitigate cases where the OKN was near the lower threshold of detection, and the “repeating OKN” criterion was not activated [50]. Trials that involved no OKN induction stimulus were necessarily tested for both stimulus directions because the direction of OKN was undefined in these instances.

Automated OKN detection (OKN present or absent) was performed on the gaze (OKN-G), centroid tracking (OKN-C), and motion microscopy (OKN-MMIC) signals, which were obtained on a per-trial basis. The process is summarized diagrammatically in Figure 3. Gaze was determined directly from the Pupil Neon and passed through the detection process, using gaze-specific parameters *p*. Displacement signals for the latter two C and MMIC methods (shown as dark blue processing units) were determined for individual left and right eye videos of a given trial. The same detection was used, instead of using a single set of parameters *p′* for both the OKN-C and OKN-MMIC methods.

Two additional methods were implemented, OKN-C-STEP and OKN-MMIC-STEP. In these methods: (1) the OKN-G method was first applied, and then (2) the OKN-C or OKN-MMIC results were used in case of a negative OKN-G result only. This approach focused on remediating poorly resolved OKN in the gaze signal, thereby avoiding additional computations required by the C and MMIC methods. However, by serially combining tests, we expected improved sensitivity but conversely reduced specificity, as a result of standard rules for combining diagnostic tests [50]. In Figure 3, these two methods are represented by multiplexing units (shown in light blue) that act to select the appropriate output flag based on the result of OKN-G. The STEP methods were the result of a selection process and did not require additional signal extraction/processing.

Finally, to reasonably compare all methods, the presence/absence of OKN at the trial level for the C and MMIC-based methods was found by performing a logical OR on the results for individual eyes to provide a trial-wise result. This enabled direct comparison with the OKN-G and baseline human assessment. If data for only one eye was available using the C or MMIC methods, then this eye determined overall OKN presence/absence.

### 3.8. Performance Measures

Confusion matrices were generated against the ground truth assessment for all methods. The performance of all methods was measured using the Matthews correlation coefficient (MCC), sensitivity, specificity, and accuracy. The MCC is a measure of overall performance [51] that takes into account all cells of the confusion matrix, and is recommended particularly for imbalanced datasets. The values of MCC range from −1 to 1, where −1 is a perfect negative correlation, 0 is a random correlation, and +1 is a perfect positive correlation. The sensitivity measured the ability of the method to correctly identify the presence of OKN as a fraction of all possible positive trials, whilst the specificity measured the ability of the method to correctly identify the absence of OKN as a fraction of all possible negative trials. The accuracy measured the ratio of positive detections to all detections, and more specifically, by (1-accuracy), the presence of false detections. Performance metrics were obtained by pooling across all trials for all participants and should be understood to be descriptive and valid for the data collected only. Analyses of these data were performed using MATLAB R2023a (MathWorks, https://www.mathworks.com (accessed on 1 December 2025)). Additional preliminary inferential statistical testing was performed using JASP 0.95.4 (https://jasp-stats.org/ (accessed on 1 December 2025)). Performance metrics were tested using a randomly chosen eye of each participant. However, the strength of this additional testing was expected to be limited because our testing protocol used a maximum of 12 trials only, which was a value tuned for in-community use, where time was limited.

## 4. Results

**Figure 4:**
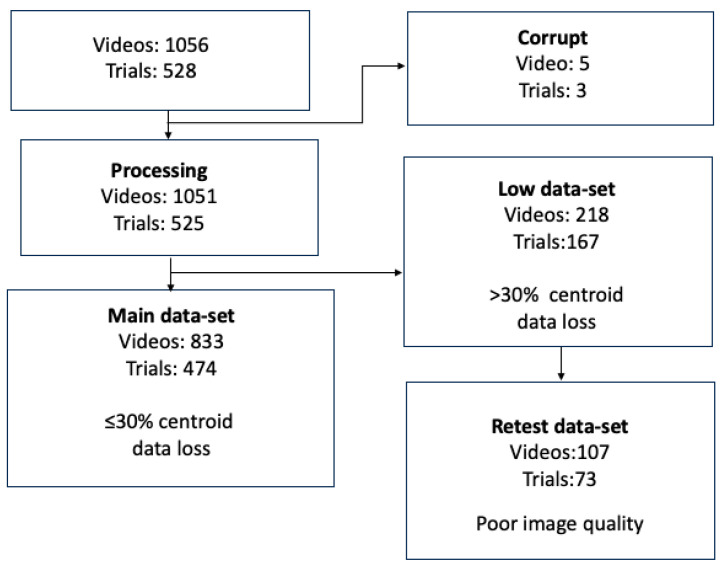
The validation pipeline. A small number of videos were corrupt and removed from processing. The centroid algorithm was applied to the remainder to sort videos into a “main” dataset (≤30% loss) or “retest” dataset in which there was significant data loss (>30%).

The process of video removal is summarized in Figure 4. A total of 528 trials (1056 videos) were manually labeled (*n* = 22 participants × 12 trials × 2 tests × 2 eyes). A total of 5 videos were corrupted and discarded. A total of 167 trials (218 videos) were ineligible for the main dataset because they contained ≥30% signal loss when processed by centroid tracking (“low data”). A total of 833 videos corresponding to 474 trials were included in the main dataset. A secondary set called the “retest” dataset comprised 107 videos drawn from the “low data” set. These videos were chosen because the failure was a result of poor pupil delineation/image processing. This set corresponded to videos from four participants (from a total possible of 192 videos = 4 participants × 12 trials × 2 eye tests × 2 eyes).

### 4.1. Results for “Main” Dataset

**Figure 5:**
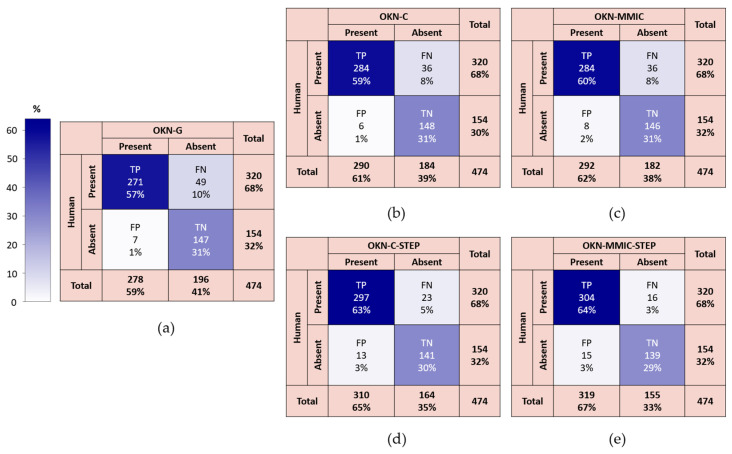
Confusion matrices for automated OKN using: (**a**) baseline gaze signal (OKN-G), (**b**) pupil centroid tracking (OKN-C), (**c**) phase-based estimation (OKN-MMIC), (**d**) centroid tracking applied to negative OKN-G tests only (OKN-C-STEP), and (**e**) motion microscope applied to negative OKN-G tests only (OKN-MMIC-STEP).

The resulting confusion matrices for the main comparison of methods are summarized in Figure 5a–e and overall statistics are summarized together in Table 1. It was found that both centroid and motion microscope methods were better than baseline (OKN-G) across the MCC (0.82–0.85 vs. 0.76), sensitivity (0.89–0.95 vs. 0.85), and accuracy (0.91–0.93 vs. 0.88) measures (see Table 1). OKN-MMIC-STEP performed the highest across these three performance metrics. However, for specificity (0.90–0.96 vs. 0.95), only OKN-C was higher, whilst OKN-MMIC was the same, and the performance of the two-step methods was lower than baseline relative to OKN-G (0.96).

Preliminary inferential statistical testing was performed across methods for MCC, sensitivity, specificity, and accuracy measures for a randomly chosen eye of each participant. The datasets were not normally distributed, and therefore non-parametric methods were used to test for statistically significant differences between methods. Friedman’s test revealed that sensitivity varied significantly across methods, X^2^_F_(4) = 17.93, *p* < 0.001, Kendall’s W = 0.22. Pairwise testing using Conover’s test with Bonferroni correction revealed that both the OKN-C-STEP (median sensitivity = 1, IQR = 0, T = 3.90, *p* = 0.002) and the OKN-MMIC-STEP (median = 1, IQR = 0.03, T = 4.20, *p* < 0.001) methods had significantly higher sensitivity than the OKN-G (median= 0.88, IQR = 0.23) method. The other methods did not differ significantly in their sensitivity. There were no significant differences between methods for MCC, specificity, or accuracy (all *p* values > 0.05). We note that these analyses are preliminary because the number of trials per participant was both small and varied across participants.

### 4.2. Results for the “Retest” Dataset

**Figure 6:**
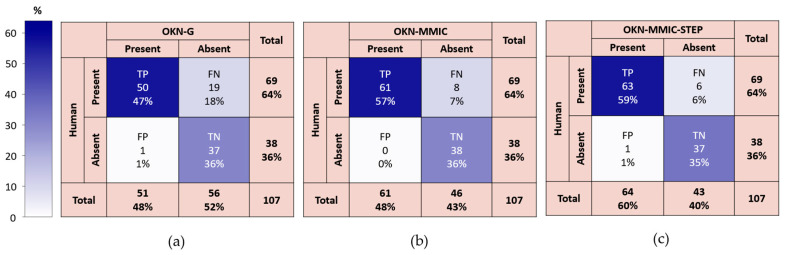
Performance metrics for the baseline method, OKN-MMIC, applied to low data and no data results from selected participant video. Confusion matrices for automated OKN using: (**a**) baseline gaze signal (OKN-G), (**b**) phase-based estimation (OKN-MMIC), and (**c**) motion microscope applied to negative OKN-G tests only (OKN-MMIC-STEP).

The confusion matrices are summarized by Figure 6a–c, and the overall performance of the three algorithms for this dataset is summarized by Table 2. It was found that both motion microscope methods had higher performance metrics than baseline (OKN-G) across MCC (0.85, 0.87 vs. 0.67), sensitivity (0.88, 0.91 vs. 0.73), and accuracy (0.94–0.95 vs. 0.81) measures. The OKN-MMIC had perfect specificity (1.00) whilst OKN-MMIC-STEP and OKN-G had the same (0.97). An inferential analysis was not performed on this dataset.

**Figure 7:**
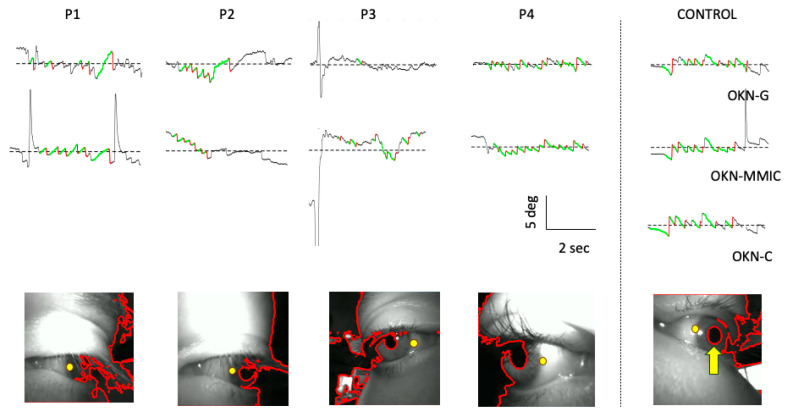
Examples of results obtained for the four participants (P1–P4) who comprised the retest group, compared with a control in the far-right column. The first row shows results for OKN-G. The second row contains data for OKN-MMIC. The third row contains data for OKN-C, but only the control produced valid data. The 1D signals are shown with detected slow phases (green) and quick phases (red) superimposed. The final row presents segmentations that reveal the source of tracking failures (P1–P4) as well as an example for the control. The reasons were: P1 = eyelashes covering the pupil, P2 = distortion of the pupil segmentation by eyelashes and shadowing linking to background pixels, P3–P4 = shadowing over the pupil created by the iris, creating a link to background pixels. Also shown are yellow dots indicating the location where data was read from using MMIC.

The first four columns of Figure 7 present signal traces obtained by the OKN-G and OKN-MMIC methods, as well as an example of a video frame with the segmentation result obtained using our implementation of centroid tracking. The examples failed to achieve robust delineation of the pupil because of the presence of eyelashes, shadows, and background lighting. The right-hand column includes the result of centroid tracking, for comparison, showing a situation in which a signal was successfully obtained. A fuller description is provided in the figure caption. The number of trials per participant was not large enough to enable inferential statistical testing.

## 5. Discussion

Our key motivation was to address the practical issue of undetected OKN found when using our Pupil Neon, ML-based eye-tracker (OKN-G). In this work, we used traditional centroid tracking (OKN-C) and a novel phase-based method (OKN-MMIC), the motion microscope method, to improve signal quality and therefore improve the detection of the presence/absence of OKN.

The results for the main dataset suggested that both methods could improve upon OKN-G, either as a standalone or in serial combination with OKN-G. The methods were compared, in the first instance, on the main dataset, which contained results without significant (centroid tracking) data loss. We found that OKN-C, OKN-MMIC, and their two-stage counterparts (OKN-C-STEP, OKN-MMIC-STEP) all showed improvements over the baseline method on three of the four performance metrics, namely MCC, accuracy, and sensitivity.

The MCC and accuracy are measures of overall performance. These results indicated that all methods were strong by MCC (0.76–0.85), and the accuracy was very good to excellent (0.88–0.93). This result included the baseline OKN-G method itself, although it had the lowest overall performance (MCC = 0.76, accuracy = 0.88). Of the methods tested, the OKN-MMIC-STEP method had the best overall performance on both MCC (0.85) and accuracy (0.92).

It is noted, however, that the two-stage/STEP methods had reduced specificity (0.9 and 0.92) against OKN-G (0.95). This is not entirely unexpected because the serial combination of methods aimed to increase sensitivity above that of the individual methods, necessarily limiting the specificity [52]. The reduced specificities that were obtained would be regarded as good to excellent, although the amount of reduction that can be tolerated will depend on the use case. It may be that further optimizations will reduce the loss of specificity, which would be the subject of further work.

A preliminary statistical analysis indicated that sensitivity was improved for both the OKN-C-STEP and OKN-MMIC-STEP methods versus OKN-G, but otherwise no significant effects were found. This analysis was conducted using performance measures that were calculated using a relatively low number of total trials per eye (a maximum of 12) that were spread across categories (TP, FP, FN, TN), and the resulting performance measures were varied as a result. The number of trials used was low because of the need to reduce the time spent collecting data, and a more comprehensive inferential analysis would be the subject of further investigations.

The results for the “retest” dataset included instances when centroid tracking failed, and results for OKN-C and OKN-C-STEP were not available. Both OKN-MMIC/OKN-MMIC-STEP and OKN-G methods returned results in this case, and the MMIC/MMIC-STEP methods produced results that were the same as or better than OKN-G. Therefore, in this case, there was a general improvement upon the baseline method, OKN-G, by using the phase.

### 5.1. Image Phase for Eye Displacement

Our work has introduced a novel approach to the measurement of eye displacement, namely the phase-based motion microscope method. Image phase is a relatively unfamiliar approach in the context of eye-tracking, although prior work showed that it could be used to amplify virtually imperceptible motions of the eye to render micro-saccadic eye motions visible [36,38]. Importantly, the phase method returned results in instances where the centroid tracking otherwise failed, as determined by the “retest” set. The method did not employ pupil segmentation, and it was robust to the lighting conditions. In this case, the results for this method also consistently improved upon those found for the baseline OKN-G method. Further enhancements to this phase method may be possible. It may be useful to consider only those levels (frequency and orientation) relevant to the motion, thereby ignoring contributions from irrelevant levels. It may be possible to optimize the location from which the displacement signal was obtained to ensure that the signal obtained was the most robust possible.

There is certainly additional scope for investigation of the image phase approach. Alternate and faster decomposition techniques have been proposed that are better suited to real-time applications [53]. Recent work by Valente et al. [54] used a phase-based video motion magnification technique [38] to amplify the presence of small-amplitude oscillations of a mechanically driven cube, after which centroid tracking and smoothing were applied. Such an approach could be readily adapted to the case of the eye. It would be interesting to investigate the potential of phase in non-head-mounted video, such as a low-cost webcam. In such applications, pupil tracking is more challenging [30], because pupil localization is substantially more difficult, and the quality of video is typically lower and more variable. Elgharib et al. showed that the motion magnification technique [38] could be used to compensate for macro-motions of the eye to reveal smaller-scale movements using video, thereby suggesting its potential [36].

### 5.2. Limitations of the Present Study

It is possible that our simple implementation of centroid tracking could be improved upon, noting here that pupil tracking has been implemented using various alternate approaches [55,56]. It is also noted that the eye camera videos from the Pupil Neon were positioned in a non-typical oblique nasal-to-temporal orientation. The lighting system of the eyeglasses combined with the background (i.e., the room in which measurements were made) resulted in “channels” of dark pixels that connected the pupil and the background, thereby making it difficult to accurately segment the pupil by thresholding. This is the situation demonstrated by the eye camera examples in Figure 5. The fact that the eye-tracker did indeed return gaze signals in these cases demonstrates the robustness of the underlying algorithms to lighting conditions. On the other hand, typical eye camera-based systems typically aim the cameras temporal-nasally, and they can often be positioned more perpendicularly, and this would alleviate the centroid tracking algorithm issue observed here.

The units of the signals determined by video extraction were in image units (pixels) rather than angular units, and there was no compensation for that. As a result, the signals obtained from C and MMIC methods were affected by the distance from the eye camera to the eye and the movement of the glasses relative to the head. The gaze signal from the baseline eye-tracker was observed to be very robust to these types of movements. Even so, we observed improvements, and specific angular calibration was not found to be essential. In this work, the parameter choices (*p*, *p′*) used for OKN detection were determined empirically. It may be possible to objectively determine parameters using optimization methods and therefore provide a more effective objective tuning of the optokinetic nystagmus detector. However, even still, the results were encouraging and indicate the potential for improvement. It is also noted that these issues may be avoided by retraining the present system on a wider range of images/eye movements or employing the methods described here to extract a valid signal from the system.

## 6. Conclusions

Motion microscopy and centroid tracking applied to eye camera video were found to reduce the number of false negative results obtained by the gaze from a commercial eye-tracker. Furthermore, motion microscopy is robust to image degradation, whereas centroid tracking is less so. It may be that motion microscopy could be used as a method to detect the presence or absence of OKN in difficult-to-adjudicate situations.

## Figures and Tables

**Table 1:** Performance metrics for the “main” dataset across all methods tested.

Method	MCC	Sensitivity	Specificity	Accuracy
OKN-G	0.76	0.85	0.95	0.88
OKN-C	0.82	0.89	0.96	0.91
OKN-MMIC	0.80	0.89	0.95	0.91
OKN-C-STEP	0.83	0.93	0.92	0.92
OKN-MMIC-STEP	0.85	0.95	0.90	0.93

**Table 2:** Performance metrics for the “retest” dataset across all methods tested.

Method	MCC	Sensitivity	Specificity	Accuracy
OKN-G	0.67	0.73	0.97	0.81
OKN-MMIC	0.85	0.88	1.00	0.94
OKN-MMIC-STEP	0.87	0.91	0.97	0.95

## Data Availability

The dataset is available on request from the authors.

## Abbreviations

The following abbreviations are used in this manuscript:

VA	Visual acuity
OKN	Optokinetic nystagmus
C	Centroid tracking
MMIC	Motion microscope
G	Gaze
OKN-G	OKN detection applied to the gaze signal
OKN-C	OKN detection applied to the centroid tracking signal
OKN-MMIC	OKN detection applied to the motion microscope signal
OKN-C-STEP	The OKN-C method applied to the OKN absent in the OKN-G results
OKN-MMIC-STEP	The OKN-MMICC method applied to the OKN absent by the OKN-G results
